# Structure, Function, and Propagation of Information across Living Two, Four, and Eight Node Degree Topologies

**DOI:** 10.3389/fbioe.2016.00015

**Published:** 2016-02-29

**Authors:** Sankaraleengam Alagapan, Eric Franca, Liangbin Pan, Stathis Leondopulos, Bruce C. Wheeler, Thomas B. DeMarse

**Affiliations:** ^1^J. Crayton Pruitt Family Department of Biomedical Engineering, University of Florida, Gainesville, FL, USA; ^2^Department of Biomedical Engineering, University of California San Diego, San Diego, CA, USA; ^3^Department of Pediatric Neurology, University of Florida, Gainesville, FL, USA

**Keywords:** structure–function, information transmission, convergence–divergence, graph theory, cortical networks

## Abstract

In this study, we created four network topologies composed of living cortical neurons and compared resultant structural-functional dynamics including the nature and quality of information transmission. Each living network was composed of living cortical neurons and were created using microstamping of adhesion promoting molecules and each was “designed” with different levels of convergence embedded within each structure. Networks were cultured over a grid of electrodes that permitted detailed measurements of neural activity at each node in the network. Of the topologies we tested, the “Random” networks in which neurons connect based on their own intrinsic properties transmitted information embedded within their spike trains with higher fidelity relative to any other topology we tested. Within our patterned topologies in which we explicitly manipulated structure, the effect of convergence on fidelity was dependent on both topology and time-scale (rate vs. temporal coding). A more detailed examination using tools from network analysis revealed that these changes in fidelity were also associated with a number of other structural properties including a node’s degree, degree–degree correlations, path length, and clustering coefficients. Whereas information transmission was apparent among nodes with few connections, the greatest transmission fidelity was achieved among the few nodes possessing the highest number of connections (high degree nodes or putative hubs). These results provide a unique view into the relationship between structure and its affect on transmission fidelity, at least within these small neural populations with defined network topology. They also highlight the potential role of tools such as microstamp printing and microelectrode array recordings to construct and record from arbitrary network topologies to provide a new direction in which to advance the study of structure–function relationships.

## Introduction

A connectome represents a map of the identity, the location of elements, and their mutual connectivity within a brain network. It is perhaps one of the first and fundamental steps toward understanding the relationship between brain structures and their functional dynamics (Sporns et al., 2005). Partial connectomes now exist for a variety of organisms including *C. elegans* (e.g., Towlson et al., 2013), zebrafish (Stobb et al., 2012), primate cerebral cortex of the macaque monkey (e.g., Goulas et al., 2014), cat (e.g., de Reus and van den Heuvel, 2013), and mouse (e.g., Mechling et al., 2014). Basic knowledge of the connectome can be used to guide development of computational models (e.g., Honey et al., 2009) or to gain insights into various neuropathologies (e.g., Alexander-Bloch et al., 2013a) including schizophrenia (e.g., Alexander-Bloch et al., 2013b), Alzheimer’s (e.g., He et al., 2008), and epilepsy (e.g., Morgan and Soltesz, 2008). Since a myriad number of dynamical states may be expressed by seemingly identical structures, any information derived from a particular instance of a connectome is alone insufficient to truly understand the relationship between the structure of that network and its functional dynamics (Honey et al., 2009, 2010; Deco et al., 2011). Unfortunately, a more precise relationship between the structural connectivity providing the skeleton over which these highly dynamic activity patterns are produced, and the flexible computational regimes that occur within that structure remain elusive (Sporns et al., 2005).

Recent advances within the *in vitro* neural-patterning technologies could provide a new way with which to conduct detailed studies of the relationship between a network’s structure and resultant functional dynamics. A wide variety of methods have been developed to direct the growth of neurons and connectivity including methods based on soft lithography (e.g., Corey et al., 1991), microstamping or micro-contact printing (e.g., Branch et al., 1998), microfluidics (Morin et al., 2006; Huh et al., 2011), and construction of structural-topologies to guide growth (e.g., Pan et al., 2011, 2015; Kanagasabapathi et al., 2012). For example, microstamp printing of adhesion molecules to promote connectivity in the form of 2D line (e.g., Feinerman et al., 2007, 2008) and grid patterns (Kam et al., 2001), such as 4D (Corey et al., 1996; Branch et al., 1998; Vogt et al., 2004, 2005; Marconi et al., 2012), 6D, and 8D (Boehler et al., 2012), have been used to investigate cell morphogenesis (Théry, 2010), the study of spinal injury and repair (e.g., Taylor et al., 2009), and transmission of information in 1D networks (Feinerman et al., 2005, 2007; Feinerman and Moses, 2006). An alternative method capitalizes on the natural tendency of neurons to follow structural features including ridges (Curtis and Wilkinson, 1997), pillars (Dowell-Mesfin et al., 2004), or application of microfluidics to guide axonal growth (Morin et al., 2006). Coupling these living but engineered neuronal structures with large-scale measurements of neural activity using multielectrode electrophysiology or advanced optical methods provides a unique platform with which specific structural topologies such as those representing brain structures can be reconstructed *in vitro* and structure–function studied in detail. Such a platform could generate important insights into the relationship between specific structural topologies, the myriad patterns of activity representing the neural dynamics that are overlaid upon that structure, and functional properties embodied by those networks (Maccione et al., 2012).

In this study, we describe the creation of neural structures roughly based on one of the guiding principles of cortical-functional organization: the integration and segregation of information (Zeki and Shipp, 1988; Tononi et al., 1998). Specifically, a key structural property crucial for integration and segregation is that of the convergence of connections into (in-degree) or divergence of connections emanating out of each area (out-degree) (Sporns et al., 2004; Négyessy et al., 2008). Since recurrent networks represent a substantial part of connectivity, it has been proposed that correlations between firing in spike trains originate to a large degree in the convergence and divergence of direct connectivity and presence of common inputs (Shadlen and Newsome, 1998) and must therefore strongly depend on connectivity patterns (Kriener et al., 2009). Moreover, correlated inputs through convergent or divergent connections may also play a prominent role in neural and population coding (e.g., Shamir, 2014). Recent theoretical work has reinforced recurrent connectivity as an important factor in correlation dynamics (e.g., Ostojic, 2014). In fact, there are now a number of experimental studies that support this idea (Kazama and Wilson, 2009; Cohen and Segal, 2011; Smith and Sommer, 2013). Yet others have shown relatively small correlations with weak common input effects and even suggested a mechanism of active *decorrelation* (Ecker and Tolias, 2014).

To study the effect convergence on network functional dynamics, we assess the functional connectivity and fidelity of information transmission (e.g., Germano and de Moura, 2006; Czaplicka et al., 2013) in these networks. Functional connectivity refers to the extent to which activity measured at different locations within a network is correlated (Friston, 1994), and a variety of methods are now available to estimate the functional connectivity including cross-correlation (Perkel et al., 1967; Aertsen et al., 1989; Poli et al., 2015), partial directed coherence (Sameshima and Baccalá, 1999; Baccala and Sameshima, 2001), directed transfer function (Kaminski et al., 2001; Eichler, 2006), Granger causality (Chen et al., 2006; Cadotte et al., 2008; Kispersky et al., 2011), and mutual information (Bettencourt et al., 2007). We use scaled cross-correlation developed by Nikolić et al. (2012) to compute functional connectivity. The nature, meaning, or how information spreads across networks varies across many disciplines physics (Karnani et al., 2009), biology (e.g., Mino and Durand, 2010; Voelkl and Noë, 2010), social science (e.g., Burt et al., 2013), and computer science (e.g., Sloot and Quax, 2012). In our study, we are interested in the fidelity of information contained within trains of action potentials as they are transmitted and reproduced from neuron to neuron in these living networks.

**Figure 1:**
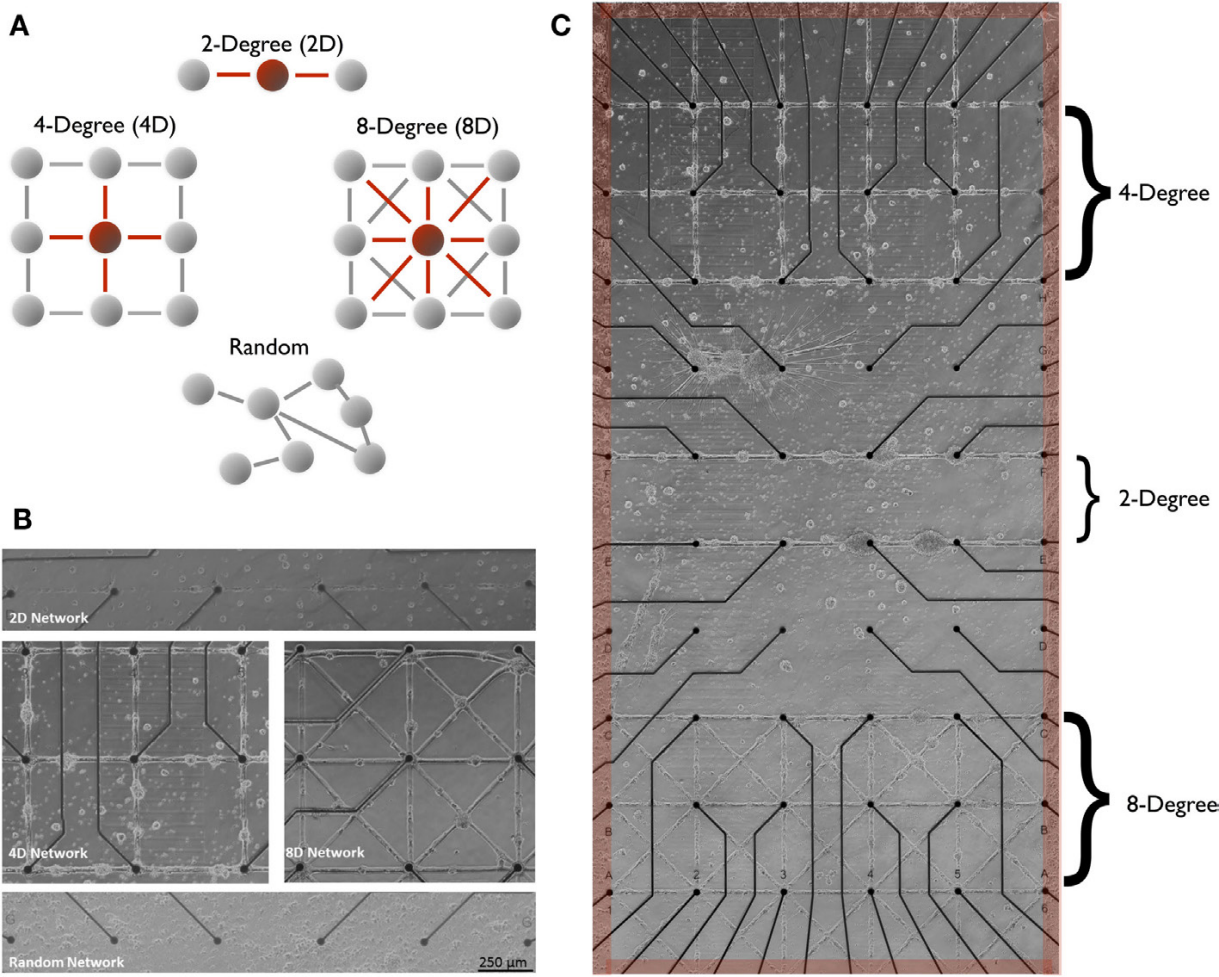
**Two-degree (linear), four-degree (city-block), eight-degree, engineered neuronal topologies measured on microelectrode arrays**. In this study, structure–function was manipulated across three distinct neuronal architectures illustrated in **(A)** that varied the number of connections from each node (node degree). Modern microstamping of cell-adhesion molecules onto microelectrode array (MEA) substrate **(B)** to create simple linear two-degree (2D) networks **(C)** (top panel), Four-Degree (city-block, 4D) grid (middle-left panel), and eight-degree networks (middle-right, 8D) seeded with rat cortical neurons. Extracellular MEA electrodes [small black circles in **(B,C)**] located under each intersection were used to measure the transmission of spike train information during spontaneous network wide bursts that originated within a surrounding pool of randomly seeded neurons [shaded red in **(B)**] and spread into each of the three architectures from the edges (red).

We first created three network topologies (Figure 1A) comprised living cortical neurons using microstamping described earlier and depicted in Figures 1B,C, each with an increasing degree of convergence. These three topologies are compared with a “random” topology in which neurons were free to connect based on their own internal properties. We then assessed the impact differing topologies have upon basic network dynamics including firing rates and network oscillations in the form of spontaneous network bursting that occurred in each network we studied. We then examined how these topologies affect information embedded within their spike trains as they are transmitted between nodes (neurons). Our primary hypothesis is that convergence modulates the effectiveness of connectivity and this effectiveness reflected in the fidelity with which spike train information is relayed during transmission from node to node across each network. We predicted that by increasing the convergence of pathways embedded within a neural structure this increase will result in more accurate transmissions at each node and hence, a more accurate reproduction of spike patterns among neurons and across the network. After estimating the underlying functional connectivity within each the four network topologies, we then assessed and compared the fidelity of information in the form of spike trains as it is transmitted from neuron to neuron across each network by measuring the similarity of spike trains reproduced by neurons embedded within each network. These dynamics produced by these structures are then compared and contrasted with results from metrics based on principles of graph theory that are now commonly used in the network analysis literature to quantify the effect of network topology upon its functional properties, and the nature and flow information as it propagates across each network of living cortical neurons.

## Materials and Methods

### Development of Patterned Networks on Multi-electrode Array

#### Preparation of the Multi-electrode Substrate and Microstamps

**Figure 2:**
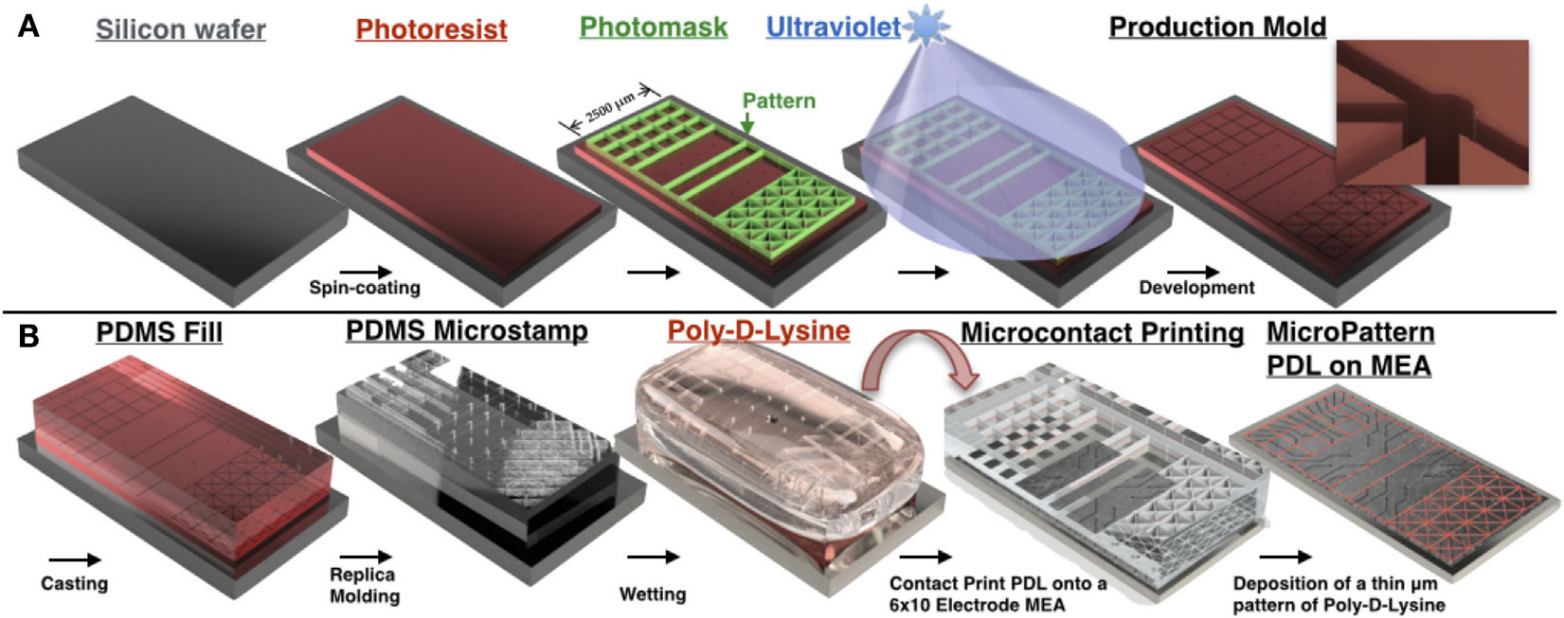
**PDMS microstamp fabrication process and procedure for microcontact printing**. Mechanical electrical machining (MEMS) technologies are used to construct a negative of a 3D structure containing micro-scale features. Photolithography is used to transfer micro-scale patterns to photosensitive materials by selective exposure to ultraviolet (UV) radiation. **(A)** A silicon wafer is spin coated with a thin film of a photosensitive material (photoresist), which is then aligned and brought in close contact with a photomask that consists of a transparent sheet with the desired pattern printed on its surface. Any desired microscale pattern can be generated by the investigator with computer-assisted design software. This is followed by exposure of the photoresist to high-intensity UV light through the photomask that protects some regions of the photoresist from UV and exposes others based on the design pattern. UV-exposed areas become soluble in a developer solution and dissolve away during the following step, termed development, thus leaving the desired microscale pattern etched into the photoresist. **(B)** Soft lithography involves fabrication of elastomeric stamps using a replica-molding technique in which liquid prepolymer of PDMS is cast against the bas-relief pattern of photoresist produced in **(A)** to generate a PDMS substrate that replicates the 3D topography of the original master. In microcontact printing, the PDMS stamp is wetted with a solution containing an adhesion promoting molecule (in our case Poly-d-Lysine), dried, aligned with the 6 × 10 grid of electrodes on the surface of the MEA, and brought in contact with the MEA surface for 30 s. Upon removal of the stamp, a pattern composed of Poly-d-Lysine is generated on the surface that is defined by the raised bas-relief structure of the stamp, and hence precisely recreates the microscale pattern of the original master. Neurons cultured over this surface will preferentially attach and extend neurites within this pattern to create a living neural network whose topology recapitulates the desired pattern. In each topology line widths were 20 μm and a pad of 50 μm in diameter was placed at each intersection [concept for figure’s design adapted from Huh et al. (2011)].

In this paper, we used the microcontact printing method developed earlier by our group to create the three patterned topologies shown in Figure 1A. A description and illustration of the procedure for the construction of molds with which to cast the microstamps, and the process of microcontact printing is shown in Figure 2. A more detailed description can be found in Boehler et al. (2012) and Branch et al. (1998).

Planar multi-electrode arrays (MEAs) were purchased from Multi-Channel Systems GmbH (Reutlingen, Germany) and consisted of 59 TiN_3_ surface electrodes and 1 ground electrode embedded within a glass substrate arranged in 10 rows of 6 electrodes shown in Figure 1C. Each electrode measures extracellular changes in the membrane voltage during the production of action potentials (spiking) of nearby neurons. Electrodes were equally spaced at a distance of 500 μm and were 30 μm in diameter. Each MEA was soaked overnight in tergazyme the day before microstamp printing (described in the following section) to remove any cellular residue from previous experiments followed by a thorough rinsing with deionized water. MEAs were then dried and treated with oxygen plasma for 5 min. MEAs were then silanized with 3-glycidoxypropyl-trimethoxysilane (3-GPS) by soaking in a solution of 3-GPS in toluene for 20 min and baked in an oven at 110°C for 40 min. 3-GPS acts as a background fill that inhibits adhesion or growth of neurons. A microstamp containing the desired pattern was then soaked in the adhesion-promoting molecule Poly-d-Lysine (PDL) and microstamped onto the surface. Unlike patterned topologies, no 3-GPS was applied for the random networks to permit growth anywhere on the surface of the MEA. MEAs were simply cleaned with oxygen plasma and coated with PDL overnight at 37°C.

#### Construction of Microstamps and Microcontact Printing

To create an SU-8 mold to fabricate each microstamp, a 10-μm layer of the negative photoresist SU-8 2010 (Microchem, Inc.) was spin-coated on silicon wafers, aligned to a mask containing the desired patterns and exposed to UV light (Figure 2A). The pattern was then developed in SU-8 developer and silanized with tridecafluoro-1,1,2,2-tetrahydrooctyl-1-trichlorsilane to assist the release of stamps from the mold in the subsequent steps. A single mold contained multiple replicates of the same pattern so that multiple stamps can be created from a single mold.

During the casting procedure, Polydimethylsiloxane (PDMS) stamps were created by mixing the elastomer with a curing agent at 10:1 ratio by weight, degassed, poured onto the SU-8 molds and allowed to cure overnight. Once the stamps were cured, the PDMS was peeled away from the molds and cut into smaller sections containing a single pattern and affixed to circular cover slips, which served as a base holding the stamp in place for positioning before printing. Stamps were soaked in 10% sodium dodecyl sulfate (SDS) solution for 15 min, rinsed with water, dried and then soaked in a 1:1 mixture of PDL and FITC-conjugated Poly-l-Lysine (PLL) for 1 h before use.

Silanization with 3-GPS causes the MEA’s surface to be hydrophobic (hence cytophobic). However, 3-GPS was cross-linked with PDL and hence enables strong adhesion of PDL to the surface creating a cytophilic pattern embedded upon a cytophobic background. The line junctions contained in each pattern within the stamp were aligned with the electrodes in the MEA using a custom built mechanical aligner and pressed against the MEA surface to enable the transfer of PDL–PLL. The transfer of the pattern was then confirmed under a fluorescence microscope. This alignment promotes the migration of cell soma toward these junctions that are conveniently colocated with electrodes for electrophysiological recording of neural activity.

#### Cortical Cell Culture

Dissociated cortical cultures were prepared from cortical hemispheres of E18 Sprague-Dawley rats (BrainBits LLC, Springfield, USA). First, the cortical tissue is suspended in 2 mg/ml Papain (Invitrogen) in Hibernate E solution (Brainbits LLC) for 20 min to digest the connective tissue. The cortical hemisphere is then transferred to a solution of Hibernate E followed by mechanical trituration in a 20-ml tube resulting in a cell suspension. The suspension is then placed in a centrifuge and spun at 1000 rpm for 2 min. This results in the deposition of neural tissue along the edges of the 20-ml tube. The supernate is removed and the tissue is re-suspended in culture medium (NBActiv4 and Penstrep, Invitrogen, Inc.). During plating, the cell suspension is dropped gently over the prepared substrate at a plating density of 700 cells/mm^2^. Cultures were stored at 37°C in a humidified incubator with 5% CO_2_ and atmosphere. Media was exchanged once every 3 days.

#### Two-Degree, Four-Degree, Eight-Degree, and Random Topologies

We investigated the effects of four network topologies depicted in Figure 1A. Each topology varies by the degree of convergence-divergence beginning with a two-degree network (Group 2D, *N* = 5 MEA cultures) composed of a simple serial chain of neurons connected by a 30-μm line of adhesion molecules. This topology, engineered with an in-degree (and out-degree) of 2, consists of a minimum of two pathways that converge upon each junction. The next topology consisted of a four-degree network (Group 4D, *N* = 5 cultures) patterned after the four corners of a city block. A junction in the 4D topology was designed to have a minimum of four pathways that converge onto it from its immediate neighbors. An eight-degree network (Group 8D, *N* = 4) included diagonal shortcuts for a total of eight potential connections. Finally, we included the more common so-called “random” network topology (Group random, *N* = 4) in which neurons are randomly seeded across the surface of the MEA and whose connectivity was self-determined. In this group, adhesion-promoting PDL was flooded across the entire surface allowing the neurons to attach anywhere and intrinsic properties of the neurons that now govern network topology (Goldberg, 2004; Rossi et al., 2007). Each of our engineered network topologies (as opposed to random) consisted of circular junctions (pads of adhesion molecules 50 μm in diameter) located over electrodes spaced 500 μm in a 6 × 10 grid shown in Figures 1B,C. Neurons in the 4D and 8D topologies were cocultured with the upper three rows of electrodes containing the 4D topology and the lower three rows containing the 8D as illustrated in Figure 1C. The 2D topology was cultured separately across along the 10 rows of electrodes using the 6 × 10 array (it is only included in Figure 1C for the sake of presentation). In the random topology, the network covered the entire 6 × 10 electrode array.

In our initial design of engineered networks, each of the patterned architectures was surrounded by, but not attached to, a pool of randomly seeded cortical neurons. The original rationale for this was that the additional neurons in the surrounding pool conditions the media with factors that promote the health and survival of nearby neurons within the patterned structure. However, we found that activity and structural connectivity within each patterned topology under these conditions was often inconsistent and frequently not sustainable as the culture matures and sometimes reported by others (Boehler et al., 2012). Our solution was to surround each topology on all sides with a much larger pool of spontaneously active neurons as before, but now each of the engineered architectures (i.e., 2D, 4D, and 8D) were anchored along the edges to the surrounding pool. The edge of the pool of neurons is highlighted in Figure 1C with a red border. By attaching this dense pool of neurons in the surrounding area to each topology at anchor points along the left and right hand edge, activity emanating from the larger outside pool could enter into and be transmitted across each of the patterned topologies.

### Data Acquisition and Analysis

#### Statistical Analysis

Our analysis of the spike trains was conducted using custom Python (Enthought 64-bit v1.2), C, C++ code, and the R statistical package (v2.15.1). All statistical tests were conducted using *t*-tests but whose results were also compared with those produced by the non-parametric Mann Whitney *U*. The family wise false discovery rate during multiple *t*-test comparisons was corrected (Benjamini and Hochberg, 1995). Probabilities below 0.05 were considered significant. All error values including error bars in the figures represent the mean ± SEM.

#### Spike Detection and Spike Sorting

Extracellular signals from the neurons were recorded for 5 min beginning on day 14 after plating using a Multichannel Systems 1060BC (Sampling rate 25 kHz, bandwidth 8–10 kHz). Raw signals from each electrode were stored to disk using MC Rack software for later offline analysis. Spikes (action potentials) were detected by the crossing of a threshold set at five times the SD (5σ) of noise level. Electrodes with firing rates less than 0.1 Hz were discarded. Spikes were then sorted into single units for each electrode using the surrounding ±1 ms of each spike’s waveform using the first three components from principle components analysis (PCA) followed by unsupervised k-means based on the KlustaKwik algorithm (Kadir et al., 2014). The average number of sorted units (putative neurons) detected per electrode following spike sorting was similar in the 2D (1.5 ± 0.1), 4D (1.8 ± 0.4), 8D (1.9 ± 0.5), and random topologies (1.6 ± 0.04) (*p* > 0.38). Throughout this paper, we will refer to each sorted neuron as a node for convenience.

#### Burst Detection

Neurons that are cultured *in vitro* spontaneously begin producing action potentials as neurites extend and connectivity expands. As these networks mature, this early activity will gradually coalesce into and form short spontaneous network wide bursts of action potentials. Spontaneous bursts were detected on individual electrodes using the method described by Wagenaar et al. (2005). Briefly, each spike train produced by each channel was spike sorted and searched individually for burstlets (sequences of at least four spikes with inter-spike intervals less than a threshold set to 25% of that neuron’s inverse average spike rate). Burst duration was estimated as three times the SD of ISI values from qualifying spikes. Bursts with durations less than 10 ms were discarded. Peak firing rates during bursts and the times at which firing rates reached their peak were estimated from the location of this peak within a smoothed (5 ms Gaussian blur) histogram of spike counts (1 ms bins).

#### Measures of Functional Connectivity

We computed the scaled cross-correlation analysis (SCA) (Nikolić et al., 2012) to estimate function connections among the sorted neurons measured at each electrode. Traditional cross-correlations are susceptible to distortions due to non-stationarities produced by bursts of neuronal activity that is typical of these cultures. Like traditional cross-correlation, SCA also computes a cross-correlation among binned spike times. However unlike traditional cross-correlation, SCA computes a Pearson correlation coefficient over a moving but short temporal window during which the two spike trains can be considered quasi-stationary. In this study, the temporal window for SCA was 20 ms with bins of 1 ms and computed over a 100 ms temporal window to detect short- and long-distance connections. The criterion for significance of any peak in the cross-correlation was three consecutive bins containing significant Pearson correlations as described in Nikolić et al. (2012). Any peaks whose time lag represented conduction velocities outside the range of 0.1–0.8 m/s (Patolsky et al., 2006) were discarded (Garofalo et al., 2009). Network analysis was generated using the Network X python package freely available from the Los Alamos National Laboratory.

#### The Fidelity of Transmission of Information across Each Topology

To measure fidelity with which spike trains are transmitted among neurons, we applied a classic cost based measure by Victor and Purpura (e.g., Victor, 2005) suitable for the analysis of spontaneous activity produced by these living networks. The Victor–Purpura metric is a metric that calculates the distance or *dissimilarity*, *D_*v*_*, between two spike trains as the cost of transforming one spike train into the other following a series edit operations (insertion, deletion, or temporal shifting). While the operations of insertion and deletion have a fixed cost of 1, the cost of shifting a spike in time, Δt, is *q*|Δt| where *q* is a parameter that adjusts the cost per unit time for that shift. This parameter essentially varies the relative temporal scale at which the metric assesses similarity at a temporally coarse scale corresponding to a rate-based modulatory code in which the cost of temporally shifting a spike is low (80 < *q*^−1^ < 200 ms), to an analysis focused on any fine grained temporal information contained with more precise spike timing where the cost of shifting any spike is high (2 < 1/*q* < 20 ms). Unlike other measures such as mutual information (Cover and Thomas, 2006) that sometimes require spikes to be placed into temporal bins prior to calculation (e.g., Ross, 2014), Victor–Purpura’s metric does not require binning, can accommodate propagation delays inherent in living neural networks due to the flexibility edit/cost operations, and can be normalized by the intrinsic firing rates of neurons during each pairwise comparison between neurons for comparison across experimental groups [*D_v_* = *D_v_*/(*n*_1_+*n*_2_), where 0.0 < *D_*v*_* < 1.0] (Kreiman et al., 2000).

The normalized dissimilarity estimate, *D_v_*, ranges from 1.0, indicating highly *dissimilar* spike trains, to 0.0, in which spike trains are nearly identical. For the purpose of clarity during the presentation of our results, *D_*v*_* is expressed in terms of its converse, *similarity* (λ), where λ = (1.0 − *D_*v*_*) which refer to as “fidelity” for ease of discussion. Fidelity (λ) ranges from 1.0 (similar spike trains reflecting high fidelity in the reproduction of spike timing during transmission from neuron to neuron) to 0.0 (dissimilar or low fidelity).

#### Characterization of Functional Network Topology

The results from our functional connectivity metric based on SCA and subsequent spike sorting were first used to construct a weighted multigraph for each MEA (a graph permitting weighted and potentially bi-directional connections between node pairs). Connections were weighted according to the peak Pearson correlation coefficient, *r*, from SCA (0 ≤ *r* ≤ 1.0). Many network measures remain unavailable for directed multi-edge graphs. In those cases where a directed version of a metric was not available an equivalent *undirected* graph that maintained the same number of nodes and presence of a connection between nodes was created. In the event of reciprocal connection between a node pair, the connection in the undirected graph was represented by a single link weighted according to the average weight of the two reciprocal edges.

The four metrics commonly used to characterize networks are the node degree, the characteristic path length, the clustering coefficient, and mixing characteristics of connectivity known as Assortativity. For review of these measures, see Boccaletti et al. (2006). In this study, each of these metrics are applied in order to assess the properties of each network’s actual topology elucidated by our functional connectivity metric and quantify how these properties affect the fidelity of spiking during transmission from neuron to neuron. We adopted the naming convention within the graph theoretic literature and referred to each sorted unit (putative neuron) as a “node” in the network, which is different than the nodes or junctions created within the patterns by the microstamping procedure. Node degree (*k*) represents the total number of connections for each node (neuron) computed over the original directed or undirected graphs in which the presence or absence of a connection was binary (e.g., the network illustrations provided earlier in Figure 1A are undirected). Node degree can be further subdivided into the number of arriving connections (in-degree), analogous to the concept of convergence, and out-degree, analogous to the concept of divergence. However, the overall pattern of those edges (i.e., who connects to whom) may also play a role in the fidelity of transmission. Our second metric measures the characteristic shortest path length (*L*) between nodes. Path length represents the average number of nodes among the shortest paths between all possible pairs and is one measure of a network’s efficiency. The path length *L_*ij*_* between two neurons is defined as a minimum number of connections (and hence nodes) through which the action potential must travel to get from one neuron to another. Like the children’s game of telephone, a message transmitted through multiple layers will likely become degraded. According to this analogy, as the number of nodes that must be traversed to reach a distant node increases, the fidelity during the reproduction of that spike train on that distant node may be degraded. In this experiment, we compare information across topologies that vary in convergence, that may affects the rate of that decay. Our second prediction is that the fidelity of information contained within spike trains should decay more with increasing propagation distance (i.e., number of neurons traversed) in network topologies with higher convergence–divergence compared to those with lower convergence–divergence. The clustering coefficient (*C*), introduced by Watts and Strogatz (1998), and average-weighted clustering coefficient (Saramäki et al., 2007) are a measure of the local network topology surrounding each node.

## Results

### Structural Characteristics and Basic Functional Dynamics

**Figure 3:**
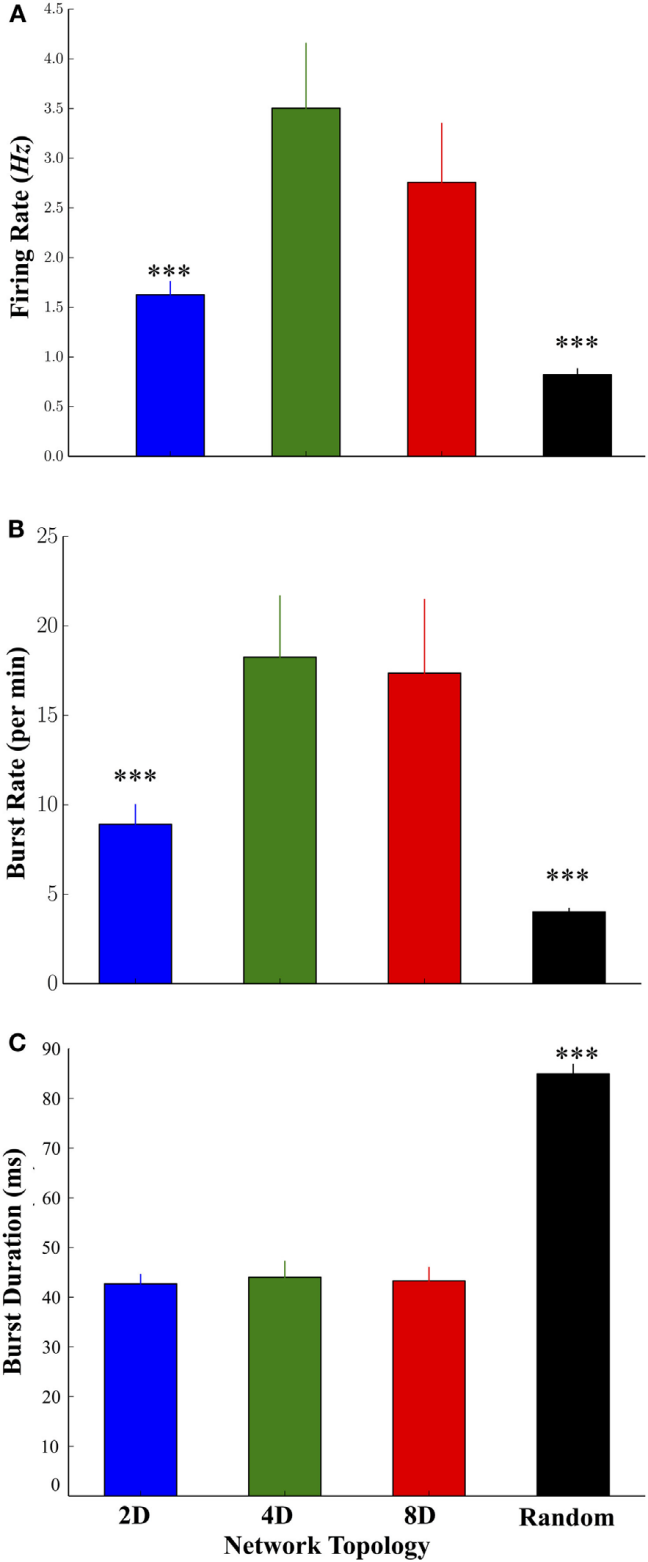
**Comparison of the firing dynamics during spontaneous neural activity across the four topologies**. **(A)** The degree of convergence within the 2D, 4D, 8D, and Random network topologies produced significant changes in the dynamics of spontaneous activity including higher firing rates in the 4D and 8D networks, relative to 2D and Random networks. **(B)** The rate at which spontaneous bursting occurred was also higher in the pattern topologies relative to Random networks. Increase burst rates paralleled a decrease in the average duration of each burst **(C)** in 2D, 4D, and 8D topologies relative to Random networks in which neurons were free to connect based on their own intrinsic properties (*p < 0.05, **p < 0.01, ***p < 0.001).

Increasing the convergence of pathways in the 2D, 4D, and 8D topologies resulted in significant changes to the highly dynamic activity patterns of neurons occurring within those structures. Mean firing rates among neurons in the 4D and 8D networks were higher relative to 2D network topologies while the lowest rates were observed in the Random topology, and no significant difference between 4D and 8D (Figure 3A) was observed. Network oscillatory behavior consisting of spontaneous recurring network wide bursts of activity occurred more often in the 4D and 8D topologies (Figure 3B) relative to 2D networks that burst more often than Random cultures where we observed the lowest burst rates overall. The lower average rate of bursting in the Random topology paralleled a much longer average duration of those bursts within that topology compared to others, which did not significantly differ from each other (Figure 3C). The average firing rate of neurons during those bursts were higher in the 2D, 4D, and 8D topologies relative to Random (19.3 ± 3.2, 26.5 ± 7.8, 16.5 ± 2.3, and 7.5 ± 0.6 Hz, respectively, *p*’s < 0.001), but did not differ among the patterned network topologies (i.e., 2D, 4D, 8D). Changes in topology also did not influence peak firing rates of neurons achieved during each burst event (85.2 ± 3.2, 80.7 ± 3.3, 83.0 ± 5.3, 89.2 ± 2.7 Hz, in 2D, 4D, 8D, and Random topologies, respectively, *p* > 0.05). There were no significant differences in the percentage of active vs. inactive electrodes containing neural activity (i.e., electrodes with spike rates > 0.1 Hz before sorting) among cultures in the 2D (43.4 ± 9.7%), 4D (67.7 ± 9.4%), 8D (65.3 ± 8.3%), and Random (67.37 ± 11.2%) topologies (*p*’s > 0.29).

### Comparison of the Fidelity of Information during the Reproduction and Transmission across Each Network Topology

**Figure 4:**
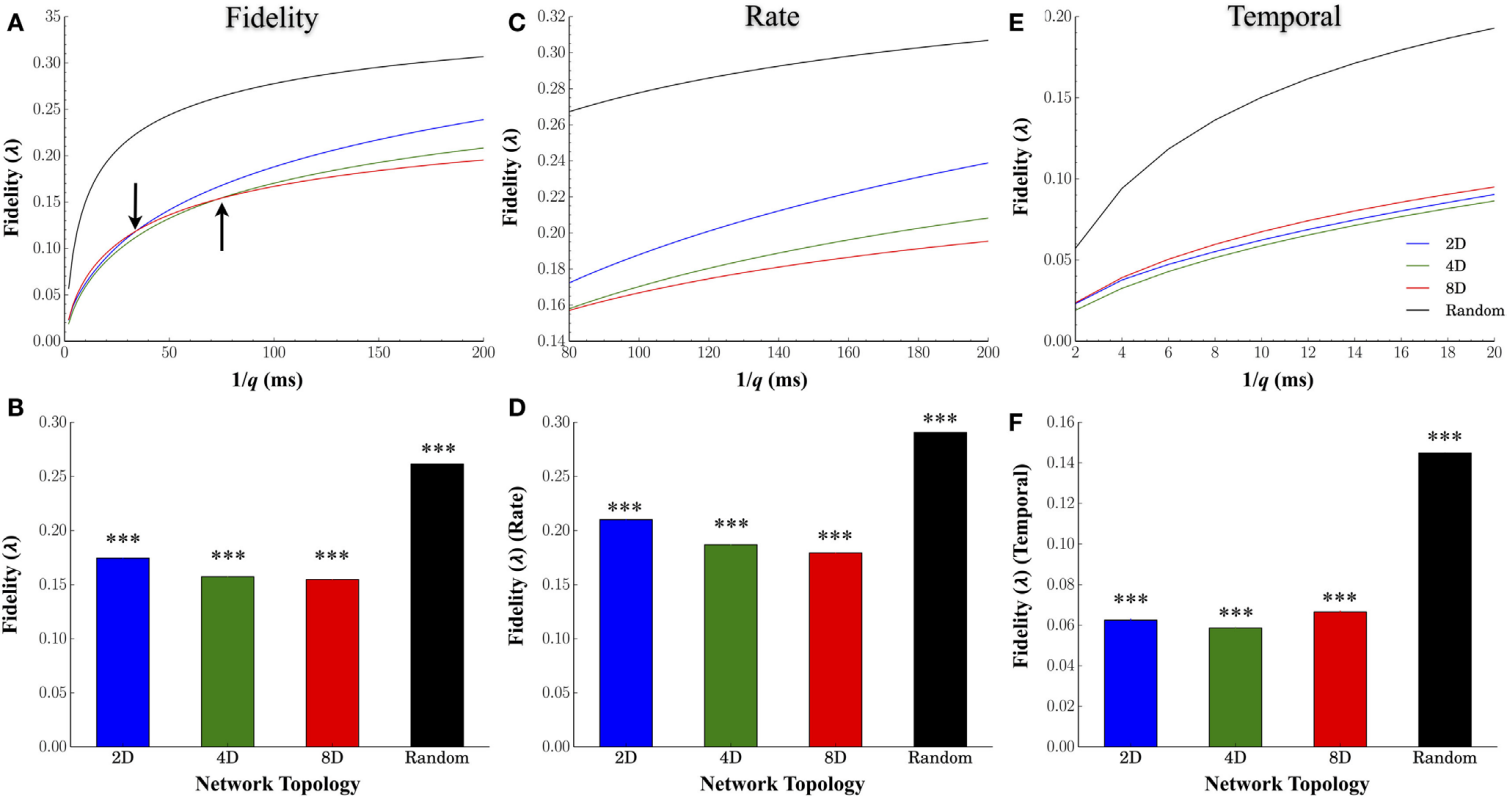
**Effect of network topology on transmission fidelity**. To assess the nature and fidelity of information embedded within spike trains during transmission between layers, we calculated a common spike train similarity metric by Victor–Purpura’s cost based metric. **(A)** Mean fidelity scores at each value of cost parameter, q, and **(B)** overall. Fidelity scores associated with rate based **(C,D)** and rate-based coding **(E,F)**. In each group, fidelity was higher overall at more coarse rate based scales (top right of each plot) rather than more precise temporally based scales (clusters of points at the bottom left of each plot).

Figure 4A plots the average fidelity relative to the cost parameter *q* (expressed in milliseconds), the average for each topology across all values of *q* in Figure 4B, and by time-scales of *q* associated with rate (*q*^−1^ ≥80 ms, Figures 4C,D) and temporal coding (*q*^−1^ ≤ 20 ms, Figures 4E,F). Manipulating the degree of convergence–divergence among topologies resulted in significant changes in the fidelity with which spike-trains were transmitted between nodes. One major effect was the enhanced fidelity at scales associated with a rate based modulatory code compared with finer temporal scales (compare fidelity scores between right and left half of each plot in Figure 4A). A second major effect was the high performance (high fidelity of information transmission) of Random topologies. Of all the network topologies we tested, the Random topology in which neurons are free to connect based on their own intrinsic properties that produced the highest average fidelity estimates compared to any other topology or time scale (*q*) we assessed (Figures 4B,D,F). At rate based scales of *q*, the group with the *least* convergence (i.e., Group 2D) produced the *highest* estimates of fidelity relative to the 4D followed by the 8D patterned topologies (Figure 4D). However, the superior fidelity estimates in the 2D, 4D, and 8D topologies were entirely dependent on the time-scale (*q*) at which fidelity was assessed. At scales of *q* associated with a rate-based coding (Figures 4C,D), the fidelity for 2D networks was superior to 4D networks. In fact, the fidelity at rate-based scales in the 4D networks was also higher than 8D networks. However, at finer time scales associated with temporal coding of information in spike trains (*q***^−^**^1^ < 20 ms) (Figures 4E,F), the 8D networks were *superior* to 2D and 4D networks. Interestingly, this reversal of fidelity scores in the patterned topologies appeared to occur at time-scales reminiscent of those associated with the duration of burst events reported earlier in Figure 3C (indicated by arrows in Figure 4A).

### Structure–Function Relationships in Engineered Networks

#### Connection Probabilities and Distance

**Figure 5:**
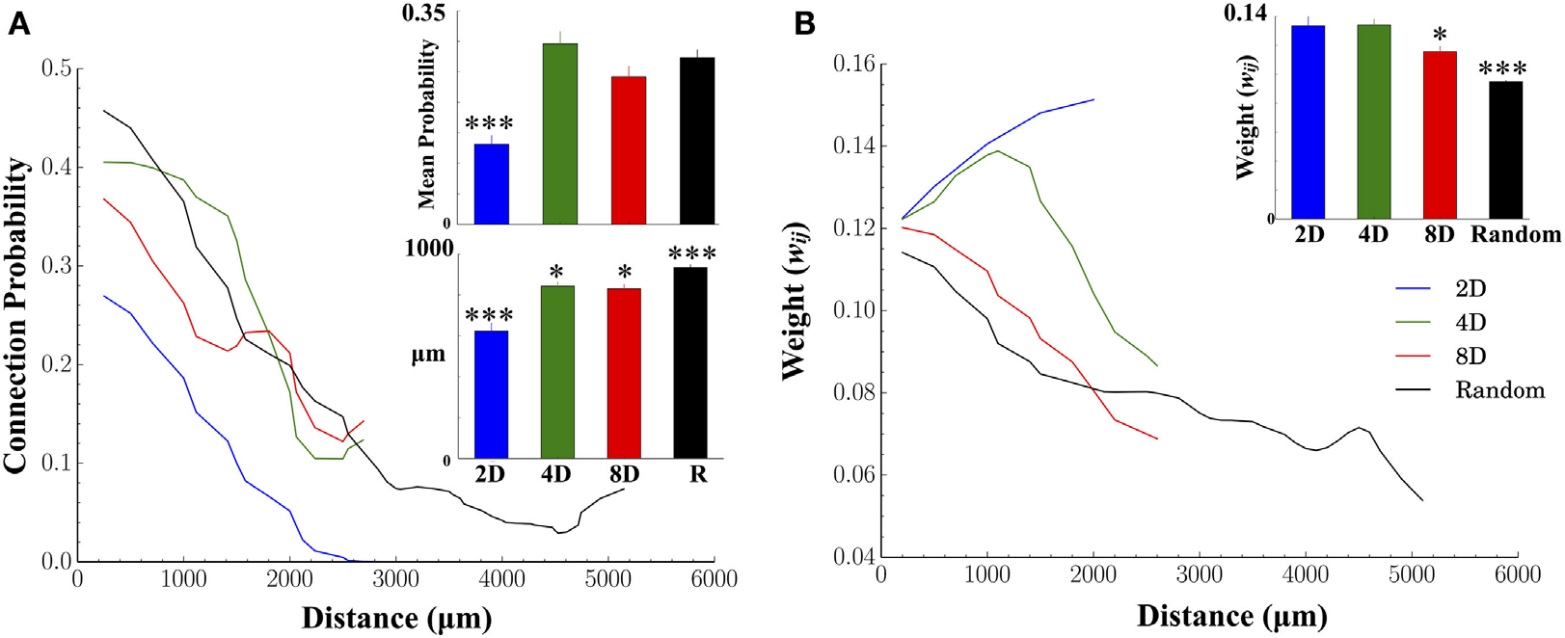
**Structural connectivity relative to physical distance and connection weights between nodes**. In both human and animals, connectivity is often constrained by biology to be distributed across finite distances where the probability of forming connections decreases the more distant the postsynaptic target is. **(A)** As expected, we found significantly fewer connections were formed with increasing physical distance for each network topology. In the 2D topology, mean connection probabilities were significantly lower than other topologies. Unlike other groups, in the 2D topology, connectivity was forced to travel along a straight line. Typical connection distances of those connections (for connections less than 2500 μm for comparison among groups) were significantly higher in Random, 4D and 8D, relative to 2D. **(B)** Connection weights were also weaker for connections at increasing distance. The largest weights were found in 2D networks followed by 4D, 8D, and finally Random topologies (inset upper right) while the distribution of weights (lower left) appear exponential irrespective of topology.

Figure 5 displays the results of two fundamental characteristics of the structural morphology estimated from information derived from our functional connectivity estimates. These were connection probability and connection weights and each are shown relative to the Euclidean distance between the electrode locations associated with functionally connected node pairs. There was a decrease in connection probabilities with increasing distance in each of the topologies we tested (Figure 5A, line plots). In addition, manipulation of the network topology had a significant impact on the likelihood of forming connections over those distances. Of all patterned topologies nodes within the 2D topologies were least likely to form a functional connection when connectivity was constrained to a single pathway (c.f. mean connection probability Figure 5A, inset upper panel and blue line) compared to the 4D, 8D, and Random topologies which did not differ from each other. As expected, the likelihood of observing a functional connection fell off rapidly with increasing distance in the 2D, 8D, and Random topologies (Figure 5A line plots). Interestingly, nodes in the 4D topology were almost equality likely to form a connection among their immediate neighbors (up to approximately 1000 μm or two nodes distant at 500 μm electrode spacing) before probabilities fell off rapidly to levels similar to 8D networks.

There were also significant differences between topologies in terms of the mean length of all functional connections (Euclidean distance in micrometer, Figure 5A bar graph lower panel). The mean length was significantly shorter in 2D relative to 4D and 8D topologies, and slightly shorter in 8D topologies compared to 4D. The longest average functional connection lengths were observed in the Random topology in a neuron’s growth was unconstrained and could cover the entire 6 × 10 electrode array.

An increase in the physical distance between nodes was also associated with a decrease in the connection weight between those nodes (In our functional connectivity metric connection weight is estimated as the peak Pearson correlation coefficient, *r*, where 0 ≤ *r* ≤ 1.0). Figure 5B plots the average connection weights between functionally connected nodes as a function of the physical distance between those nodes and average weights (bar graph inset). Like connection probabilities in Figure 5A, connection weights in Figure 5B generally appear to decrease with distance with the exception of the 2D and 4D topologies. In 4D topologies connection weights actually *increased* over short distances up to approximately 1000–1500 μm (distances that parallel the increased connection probabilities observed in Figure 5A) before declining. In the 2D topology, the average connection weight *increased* throughout the entire length of the line pattern to which connectivity was confined. Connection weights were also significantly higher overall (Figure 5B, inset upper right) in 2D and 4D networks compared to 8D and Random topologies.

Reciprocal connections (nodes with a two-way bi-directional communication path) are a feature of connectivity often reported in cortex (Holmgren et al., 2003; Song et al., 2005), between brain areas (Song et al., 2011), occur significantly more often than statistical comparisons to random network equivalents (Sporns, 2000), and observed by others within the 4D network topology used in this study (Vogt et al., 2005). More importantly, there are reports suggesting that the presence of reciprocal connections may be highly influential on the process of network communication in biological networks (e.g., Tononi and Sporns, 2003). In our study, reciprocal connections were observed among nodes in each of the topologies we created. They were however, relatively uncommon appearing among less than 10% of all connections. Manipulation of the network topology did have a significant effect on their likelihood. There were significantly fewer reciprocal connections as a percentage of all connectivity in 2D networks (0.6 ± 0.2%) compared to 4D (7.8 ± 1.2%), followed by 8D (3.5 ± 0.8%) and Random (3.4 ± 0.7%), which did not differ from each other (*p* > 0.90). These reciprocal connections also tended to occur more often at relatively short distances (623.1 ± 40.7, 842.5 ± 22.1, 829.9 ± 22.1, 934.1 ± 15.1 μm in 2D, 4D, 8D, and Random topologies, respectively) with over 80% of all reciprocal connections occurring within a span of 1000.0 μm (2D), 1414.2 μm (4D), 1118.0 μm (8D), and 2061.5 μm (Random) of each neuron (i.e., approximately within a space spanning approximately two electrodes on the MEA) (results not shown). Interestingly, the likelihood of observing a reciprocal connection was almost linear with distance. In each topology, the probability of observing a reciprocal connection changed very little within the span of two electrodes (i.e., approximately the first 1000 μm) at which point the likelihood of observing a reciprocal connection fell precipitously (data not shown) irrespective of network topology. This limited extent suggests that whatever effect these connections have upon the network, that effect is local to a node’s immediate neighbors. Finally, over 43% of nodes in 4D and 8D and 90% of nodes in Random topologies that were within a reciprocal connection were also among the high degree nodes possessing greater than 10 connections. In fact, the average node degree of reciprocal nodes was 29.26 ± 4.51 in 4D, 28.06 ± 4.77 in 8D, and 36.83 ± 5.27 in Random topologies and as mentioned in the following section, these high degree nodes were also associated with some of the highest fidelity estimates, which are observed in this study.

There were clear differences between topologies in the likelihood of forming functional connections, the weight of those connections, and the distance between functional connections. We next asked whether any of these differences influence the fidelity of transmission. For example, longer physical distances might be associated with greater decay in transmission fidelity within these cultures. Similarly, stronger connection weights might be associated with greater fidelity compared with weaker functional connectivity. However, there was little evidence of any correlation among physical distance and average fidelity between node pairs in Group 4D (*r* = −0.027*, p* = 0.4), 8D (*r* = −0.005, *p* = 0.9), Random (*r* = −0.026, *p* = 0.108) and only marginal correlation in 2D (*r* = −0.147*, p* = 0.03). Conversely, connection weights were weakly correlated with fidelity estimates in the 2D (*r* = −0.288, *p* = 0.001), 4D (*r* = −0.194 *p* = 0.001), 8D (*r* = −0.201, *p* = 0.001), and Random topologies (*r* = −0.212, *p* = 0.001).

#### Path Length and Fidelity

**Figure 6:**
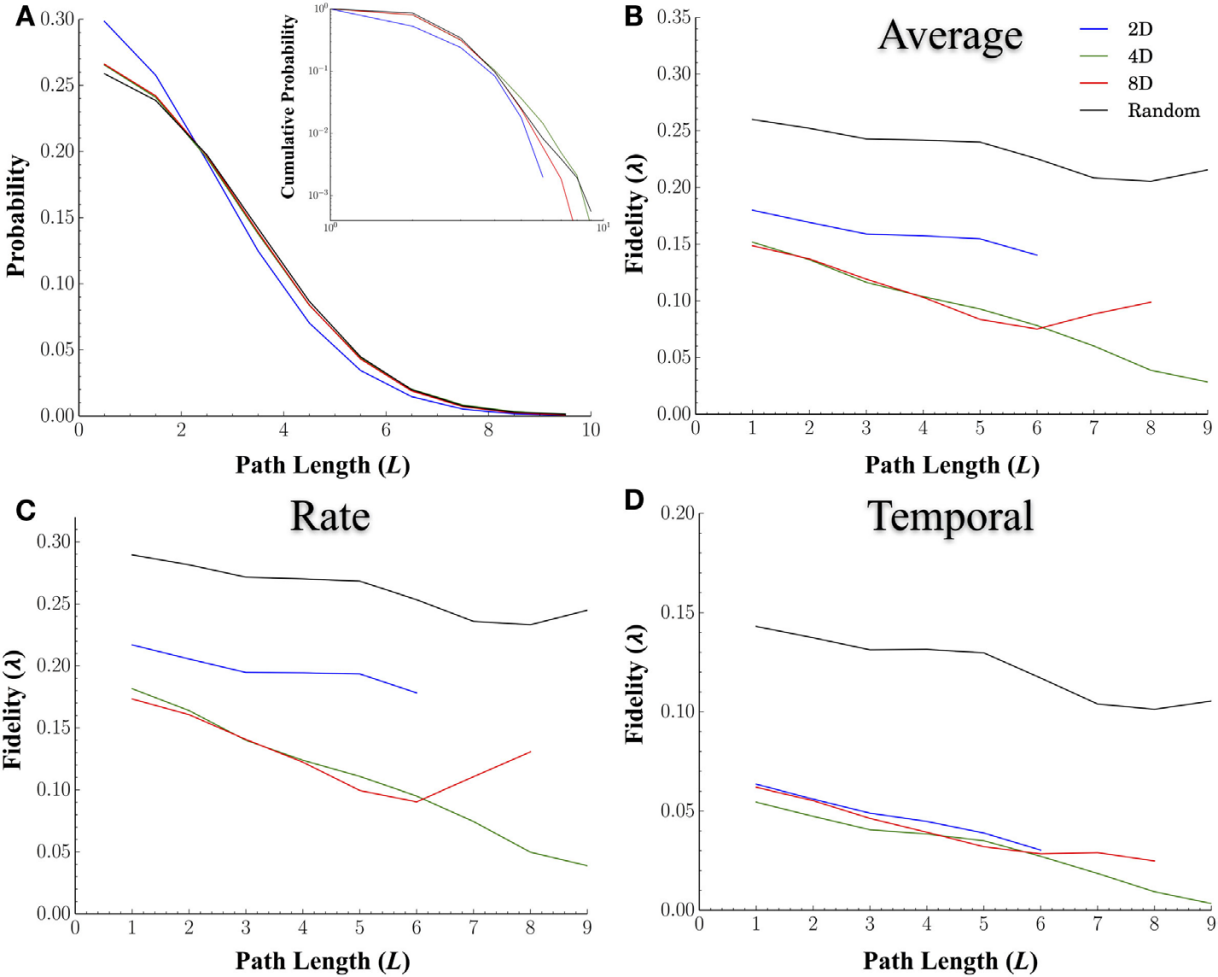
**The fidelity during neural transmission decreases with increasing number of nodes that must be crossed to reach other nodes**. We hypothesized that longer path lengths and hence, more intermediary nodes, may degrade the fidelity at which spike trains are transmitted between nodes. **(A)** displays the probability (main panel) and cumulative distribution (inset) of path lengths for each topology. We computed the similarity overall **(B)**, and at rate based scales **(C)**, and temporal scales **(D)**, between nodes relative to the shortest path length connecting the two. In general the fidelity did decrease with increasing number of nodes that must be crossed. Once again Random topologies transmitted spike trains with the highest fidelity overall and at both rate and temporal scales. While 2D networks were superior at rate-based scales **(B)** relative to 4D and 8D, this apparent advantage disappeared at temporal scales. We also hypothesized that increasing network convergence may lead to reduced rate of decay in fidelity with distance (i.e., a more shallow slope compared to Random Topologies). The idea being that convergence may produce fidelities that are more robust over longer distances. However, our results based on the slopes of path length relative to fidelity did not support this.

In a network analysis, the distance between nodes is often measured in terms of the shortest path, which measures the minimum number of nodes required to reach a destination rather than physical distance. Path length is one of the most common measures used to characterize a network’s internal structure. Unlike physical distance, path length incorporates the sometimes torturous route information must take to reach a destination (e.g., passing through multiple nodes). In fact, the characteristic shortest path length between nodes in a network may play a critical role in the transmission of information within a network by providing shortcuts with which information can travel long distances without interference or distortion from other nodes. We hypothesized that longer path lengths, and hence more intermediary nodes, should degrade the fidelity at which spike trains are transmitted between nodes. Figure 6A displays distribution of the shortest path lengths for each of the topologies. The cumulative probability distributions (inset) are also provided. Overall, the average shortest path length between nodes was smaller in the 2D networks at 1.87 ± 0.04 nodes compared to Random (2.34 ± 0.005, *p* < 0.001), 4D (2.28 ± 0.01, *p* < 0.001), and 8D (2.26 ± 0.01, *p* < 0.001) topologies that did not significantly differ from each other (*p* > 0.25).

Increasing network convergence may also lead to a *reduced rate of decay* of information as measured by fidelity over increasingly longer distances. If true, the slope of line representing the decay in fidelity over distance should be shallower in the 8D followed by 4D, and finally the 2D network topologies. We plotted the average fidelity scores by the shortest path length (*L*) between nodes (Figure 6B), for those values of *q* associated with rate (Figure 6C) and temporal coding (Figure 6D). Unlike physical distance path length was strongly related to fidelity and decreased with increasing number of nodes in all topologies. Once again Random topologies transmitted spike trains with the highest fidelity overall (Figure 6B) compared to the other topologies and did so at both the rate (Figure 6C) and temporal scales (Figure 6D). While 2D networks were superior at rate-based scales relative to 4D and 8D, at temporal scales this advantage was not apparent (Figure 6D). There was however no evidence that increasing the convergence was able to enhance or maintain fidelity over longer distances when comparing each topologies or scales we tested as slopes were similar across all topologies.

#### Node Degree

**Figure 7:**
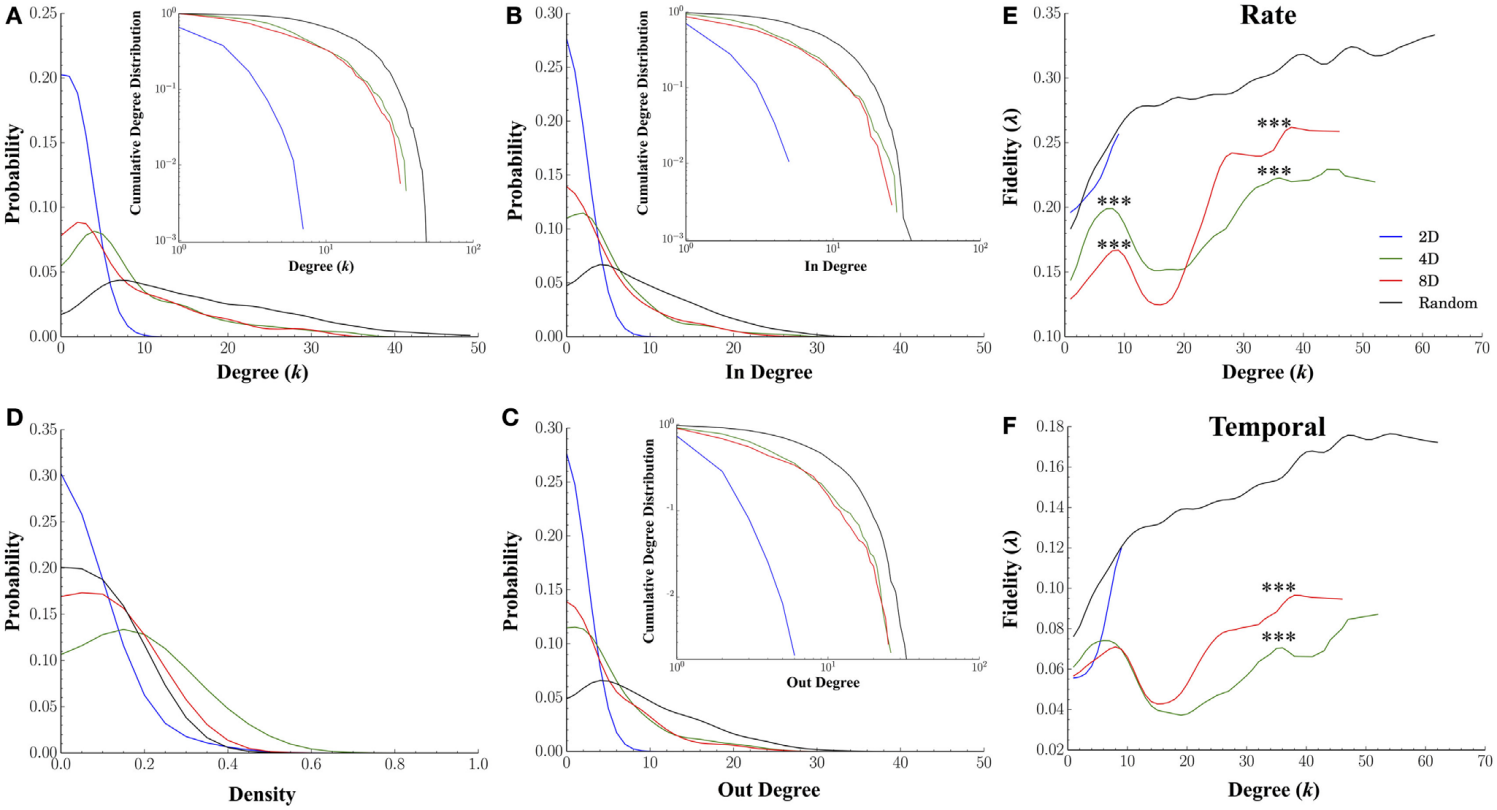
**Node degree and fidelity**. Many common network measures were affected by the structural constraints imposed by microprinting. **(A)** Node degree distribution for the 2D, 4D, 8D, and Random topologies (inset: cumulative degree distribution plotted within a log-log scale). Node degree represents the number of edges/connections per node in an undirected graph (distributions from values drawn from directed graphs were very similar in shape and magnitude). The overall average node degree was near values of 2 for the 2D, 8 for the 8D and 4D, and over 15 connections in the Random topologies. Remarkably similar distributions were obtained for the in-degree **(B)** and out-degree **(C)** suggesting the number of incoming connections were balanced with the number outgoing connections. **(D)** There were significant differences in density of node connectivity (a measure of how many connections have been established vs. the theoretical maximum) with the highest densities observed in the 4D and 8D followed 2D topologies and the lowest in Random. **(E)** plots the average fidelity associated with a rate-based neural code and **(F)** temporal-based code associated with increasing node degrees. In each topology, the fidelity associated with each node improved with increasing node degree (i.e., number of connections possessed by that node). At lower node degrees, the 4D and 8D networks produced an initial peak in fidelity that occurred at node degrees of 6 and 8, respectively, which were near original design values 4 and 8. Increases in node degree beyond those values resulted in a momentary decrease in fidelity (local minima) before once again increasing for the few nodes highest node degrees. In 2D and Random networks fidelity continued to increase up to the maximum node degree observed within these topologies.

Our first goal was to simply compute the characteristic degree of each node to determine if our microprinting method we used to guide connectivity produced the desired differences in the magnitude of convergence–divergence (i.e., number of connections as illustrated in Figure 1A). If our efforts with microprinting were successful, there should be approximately 2, 4, and 8 connections (i.e., “degrees”) for the 2D, 4D, and 8D topologies, respectfully. Figure 7A plots the degree distribution and cumulative probability distribution (inset) for each topology. Node degree distributions for each topology appeared to peak at values near those predicted by our methodology. The average degree for neurons within the 2D topology was 1.32 ± 0.05 edges per node, a value close to the desired two connections per node (i.e., neuron). The average degree for nodes in the 4D and 8D topologies were similar at 8.86 ± 0.36 and 8.06 ± 0.39 connections, respectively. Each was significantly higher than 2D networks (*p*’s < 0.001) while Random cultures resulted in the highest average node degree per neuron at 15.75 ± 0.32 (*p*’s < 0.001). Long-tailed degree distributions were observed in the 4D, 8D, and Random topologies, however, particularly among nodes with that possessed a higher number of connections. This paralleled similar distributions for in-degree (number of incoming connections per neuron, Figure 7B) and out-degree (number outgoing) connections (Figure 7C) suggesting a fairly balanced input-output relationship for each node.

The high-degree nodes (*k* > 10) were also associated with high betweenness centrality compared to low-degree (*k* < 10) members (0.04 ± 0.005 vs. 0.02 ± 0.005 in 4D, 0.04 ± 0.001 vs. 0.03 ± 0.001 in 8D, 0.02 ± 0.001 vs. 0.003 ± 0.0005 in Random topologies, respectively). This was also true of closeness centrality (0.56 ± 0.01 vs. 0.36 ± 0.03 in 4D, 0.57 ± 0.001 vs. 0.29 ± 0.02 in 8D, 0.53 ± 0.003 vs. 0.39 ± 0.02 in Random topologies, respectively), and high local efficiency (Latora and Marchiori, 2001) (0.31 ± 0.003 vs. 0.007 ± 0.006 in 4D, 0.33 ± 0.003 vs. 0.003 ± 0.009 in 8D, with the exception of Random at 0.0004 ± 0.001 vs. 0.02 ± 0.001, respectively).

Figures 7E,F plot the relationship between a node’s degree relative to its average fidelity at the rate (Figure 7E) and temporal scales (Figure 7F) from our fidelity metric. In each of the topologies we tested, an increase in a node’s degree did generally correspond to an increased transmission fidelity. 2D and Random networks nodes with larger degrees were associated with higher average fidelity at Rate (Figure 7E) and temporal scales (Figure 7F). This was also true of the 4D and 8D networks, at least up to node degrees of approximately eight connections. However, at eight connections, this local peak in fidelity was followed by a rapid decline to minimal fidelity that occurred at nodes degrees of approximately 15–20 connections before increasing once again. Interestingly, comparison of rate vs. temporal coding estimates among 4D and 8D topologies in Figure 7E vs. Figure 7F suggest that the apparent dominance of 4D for rate based coding was only at node degrees within this initial peak. Those neurons in within each network topology that were strongly connected with node degrees above 15 connections resulted in higher transmission fidelity overall. However, it was the 8D topologies that appear to dominate in terms of both rate and temporal information measured by our fidelity metric for those highly connected neurons. Comparable effects to those presented in Figures 7E,F were also observed for in-degree and out-degree (results not shown).

We also computed the node density for each network from each topology and plotted the distribution of those values in Figure 7D. Node density is measure of how completely connectivity had “filled-in” each network’s structure and ranges from 0 (unconnected) to 1.0 (fully connected or complete) and this factor alone can have profound effects on the properties of propagation within a network. For example, when a network’s densities becomes to low any activity within that network may fail to effectively propagate to every node (e.g., Kuiper, 2010), and as a result, fidelity may be compromised. We found that the density of connections in networks cultured under the 2D topology were significantly lower (mean 0.14 ± 0.03) than those in the 8D (mean 0.26 ± 0.02, *p* < 0.001) whose densities were lower than 4D (mean 0.36 ± 0.04, *p* < 0.001). Cultures in the Random condition who had the highest fidelity estimates also had the lowest average node density relative to 4D and 8D topologies (0.21 ± 0.02, *p*’s < 0.040).

#### Clustering Coefficient

**Figure 8:**
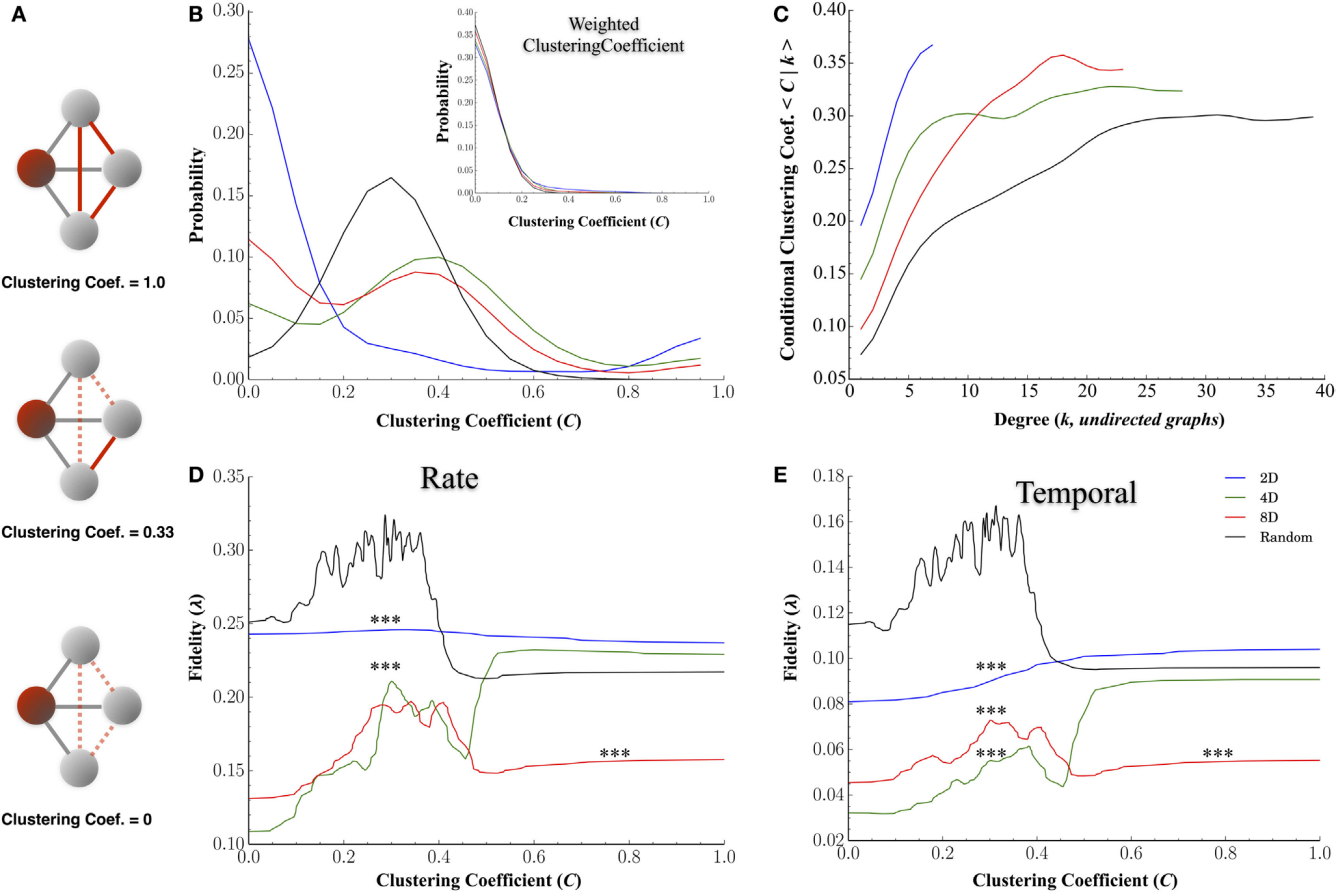
**Clustering coefficients influence transmission fidelity**. Clustering coefficient was computed from undirected graphs and is a measure of the cliquishness of a nodes surrounding connectivity and is a property known to affect the efficiency of communication. **(A)** Example of clustering coefficient calculation for the red shaded node i. Gray edges are connections from node i to three other neighboring nodes in gray, and dotted red edges are for unused possible connections. The clustering coefficient is a probability represented by the number of edges connecting neighbors of node i shown in red, divided by the total number of possible edges between neighbors of node i, shown in gray, of neighbors fully connected among themselves. **(B)** Distribution of clustering coefficients among nodes for each topology and **(C)** association between clustering coefficients and node degree (node degree among undirected graphs). **(D,E)** represent the association between a node’s clustering coefficient and transmission fidelity at rate and temporal scales with nodes it shares a functional connection with.

Figures 8B–E display the results for the clustering coefficient computed for each node and each network topology. Figure 8A plots the probability distributions for clustering coefficients across nodes for each group and conditional probability distribution dependent on node degree, *P*(*C*|*k*), in Figure 8B. Changing the topology of the network resulted in significant changes in the distribution of clustering coefficients with the highest average clustering coefficients observed in the 4D and 8D topologies (mean: 0.43 ± 0.02 and 0.53 ± 0.02, *p* > 0.08) followed by the Random (0.43 ± 0.005, *p*’s < 0.001) and 2D network topologies (0.24 ± 0.025, *p*’s < 0.001). The clustering coefficient of a node is often correlated with its degree (high node degree’s are often associated with higher clustering coefficients) and this was apparent in Figure 8B where an increased nodes degree was associated with an increased clustering coefficient. Like node degree, a node’s clustering coefficient was also associated with its transmission fidelity appearing with a peak in fidelity for nodes with coefficients near 0.3 followed by a local minima and subsequent increase for higher coefficients reminiscent of Figures 7E,F.

#### Degree–Degree Correlations among Neurons and Resultant Communication Fidelity

Communication among members of each network occurs between nodes with often heterogeneous properties. Real-world networks are correlated (Albert and Barabási, 2002; Newman, 2003a; Boccaletti et al., 2006) including biological networks (Newman, 2003b). In Figure 7, node degrees were distributed across a wide range of values and any correlations between a communicating pair of nodes with similar node degrees may have a profound influence on the quality or efficiency of that communication. Correlations between nodes properties, particularly degree-degree correlations, are perhaps one of the most studied correlations in network analysis. For example, nodes with similar degrees (e.g., high–high, low–low, etc.) may communicate more efficiently with each other than between nodes with dissimilar degrees. Conversely, the magnitude of the node’s degree may instead be the primary determinant of any efficiency during transmission of information. According to this idea, high degree nodes should then communicate more effectively than low-degree nodes irrespective of any correlation among their properties.

**Figure 9:**
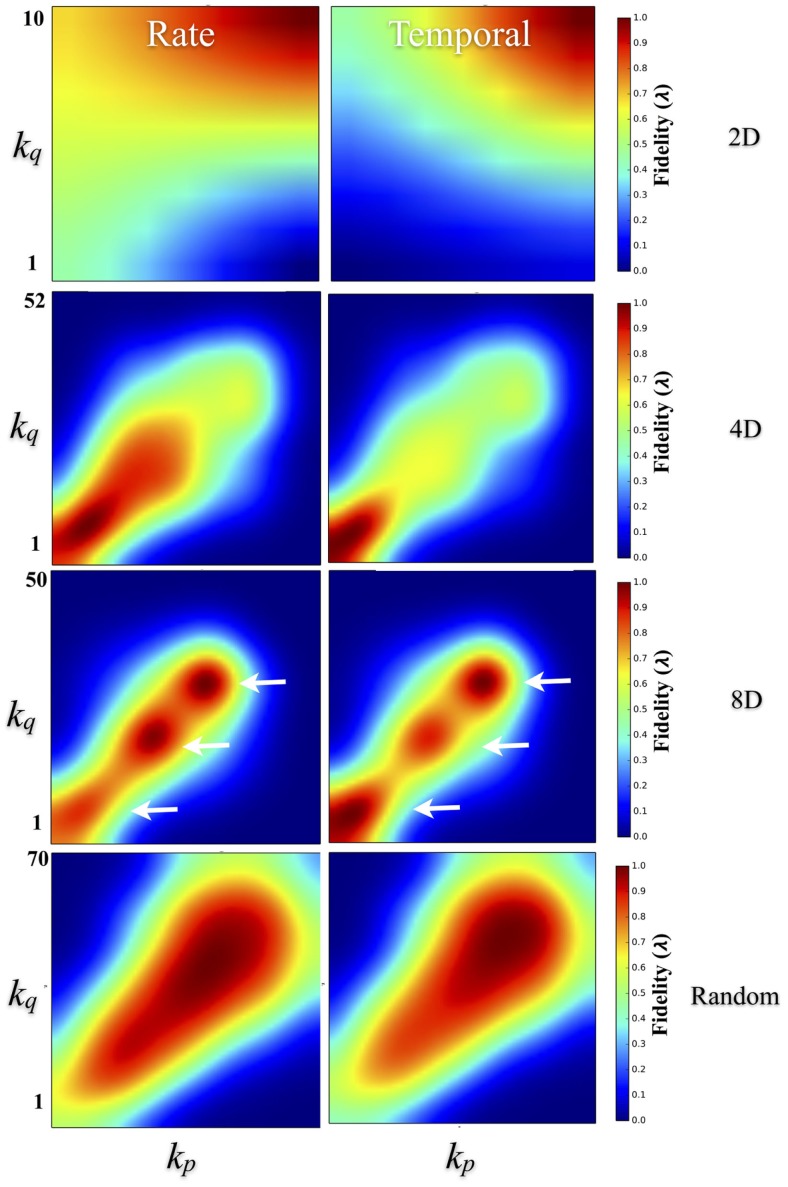
**Degree–degree correlations effect transmission fidelity**. Transmission fidelity is plotted relative to the values of the node’s degree between each communicating pair (i.e., the fidelity from nodes with degree *k*_source_ to *k*_target_) in the 2D, 4D, 8D, and Random network topologies. Fidelity is separated into rate (left column) and temporal-based scales (right column). The color scale represents fidelity from low: blue, to high: red. In the 4D, 8D, and Random networks transmission fidelity was highest during communication between nodes with similar node degrees as evidenced by the enhanced values along the diagonal. There were also non-linearities visible in the 8D and less so in 4D networks that appear as peaks (indicated with arrows) along the diagonal that occur in regions associated with node degree and fidelity observed earlier in Figures 7E,F. (Color scales are normalized within each panel from 0 to 1.0).

To quantify the effect of degree–degree correlations on fidelity, Figure 9 plots the average fidelity at rate (left column) and temporal scales (right column) by respective node degrees during communication between nodes. In the 4D, 8D, and Random topologies, the highest fidelities were measured when nodes communicated with other nodes with similar node degrees. This effect appears as a bright red appearing along a 45° line representing an interaction among nodes with similar degrees. However, the range of that correlation and associated fidelity was affected by the nature of the network topology being measured. In the Random topology, the highest fidelity estimates occurred throughout most of the range of values for node degree that we observed but were highest during communication between node pairs with similar degrees. Fidelity estimates appears highest and perhaps cluster when degree-degree values were near their peak (bulge near upper right the left and right panels). In the 2D networks, communication fidelity at temporal scales was highest between nodes with some of the highest node degrees we measured. At rate-based scales, nodes in the 2D topology produced the highest fidelity when any communication occurred with high degree nodes. Like the Random topology, in the 4D and 8D networks, the fidelity of communication was highest when node degree was correlated. However, in these topologies this relationship appeared to be somewhat non-linear with up to three peaks (indicated with arrows) appearing in 8D, and one or perhaps two peaks in the 4D topologies (right panel and left panel, respectively).

## Discussion

In any neural system, the delineation of anatomical connections is only the first step toward understanding how those connections, or their overall structure, may shape the neural activity orchestrated upon it. In this study, we employed microstamping technology to create three living network structural topologies each composed of cortical neurons. We then compare these topologies with a living “random” network analog in order to directly study the relationship between differences in a network’s structure and the changes in functional dynamics and quality of communication that results. Each of our networks were composed of living rat cortical neurons that were cultured upon a pattern of adhesion and growth promoting molecules that differed in the degree of convergence/divergence into and out of each junction. Both the structure and dynamics of among neurons that grew and developed along these patterns were indeed affected by the network topology we microstamped onto each surface. We found significant differences in structural morphology based on our functional connectivity estimates including differences in the likelihood of forming a connection, connection weights, likelihood and weights by physical distance, and by path length, node degree, and clustering coefficients. Of these, it was path length, clustering coefficients, node degree, and degree–degree correlations among communicating neuronal pairs varied by network topology and associated with a strong and sometimes non-linear effect upon the dynamics and fidelity during transmission of spike trains among each node.

### Effects on Structure on Functional Connectivity

Our first goal was to verify that the actual number of connections, measured as node degree and analogous to convergence and or divergence, were consistent with values we attempted to engineer into each patterned topology via microstamping. Node degrees among the patterned topologies did in general agreed with desired values. The exception was the 4D topology whose number of connections was closer to 8D networks rather than four connections on average. Of course, the growth of neurites is notoriously difficult to constrain *in vitro* and while not perfect, the numbers we obtained were near those we desired for each patterned topology. Why the average node degree in the 4D networks did not differ from 8D is not clear. However, one possibility is that at these small spatial scales (500 μm junction to junction spacing), the physical extent of dendrite and axon may “overrun” these patterns. For example, other laboratories have reported significant branching of neurites from single neurons using patch clamp recording methods embedded within a 4D topology nearly identical to that used here (Vogt et al., 2005). Vogt also reported that up to 45% of the time, a single neurite extending from a soma would split into multiple branches. Of those that split, 90% would branch into two neurites and 10% into three (*N* = 19 neurons). Perhaps with this much branching, it may not be surprising that the average node degree would high in both 4D and 8D topologies given the close proximity (≤500 μm) of nodes (electrode) or additional contribution of reciprocal connections (36% in the Vogt study). They also reported a threefold increase in the number of connections when comparing 4D to Random topologies which is consistent with values observed in our study in which for example, the highest average node degree was approximately 15 connections. Though node degrees were similar in 4D and 8D, there were other differences. For example, while 2D networks were least likely to form a functional connection, 4D networks seemed to preferentially attach (Figure 5A) with relatively high connection weights (Figure 5B) to neighbors with equal likelihood if they were within two nodes distant. In all topologies, however, the likelihood of forming a connection decreased distance which is consistent with *in vivo* reports (c.f. Figures 5A,B). However, neither of these measures appeared to be strongly correlated with fidelity estimates.

### Structure-Functional Dynamics

Increasing the number of inputs and therefore amount of convergence into a node should change the dynamics of neural activity at each node within the patterned topologies relative to the Random control. In fact, increasing the convergence did result in increased firing rates in 4D and 8D topologies relative to 2D and Random, and firing rates on nodes within the 2D networks were higher than Random, but there was no difference between the 4D and 8D (c.f. Figure 3A). These higher firing rates were similar to reports from other laboratories using 2D (line culture) (Chang et al., 2001), 4D (Branch et al., 2000; Vogt et al., 2005; Jun et al., 2007; Marconi et al., 2012), and 8D topologies (Boehler et al., 2012). The structural differences we implemented via microstamping were also associated with significant changes in each network’s burst dynamics. Bursting occurred more often in each of the patterned cultures compared to the Random topology and is a known effect that has been reported by our group and others in the past (Boehler et al., 2012; Marconi et al., 2012). We observed increased burst rates associated with the increasing node degrees in the 2D, 4D, and 8D topologies and reported by others (Jia et al., 2004). This is in contrast to burst durations that were longer in the Random relative to patterned topologies [and in Boehler et al. (2012)] but were not significant among the 2D, 4D, and 8D topologies or between peak firing rates during each burst (Boehler et al., 2012).

### Node Degree, Clustering, and Transmission Fidelity

According to our hypothesis, increasing the convergence of connections into and out of each junction in each topology should also increase the fidelity with which spike trains are transmitted between nodes. Our hypothesis was validated in our 2D, 4D, and 8D networks. Surprisingly of all the topologies we tested it was the Random topology that clearly produced the highest transmission fidelity among nodes (neurons) irrespective of the time scale at which this fidelity was assessed. The mean fidelity estimates in Random networks were also similar to results measured within this topology by other laboratories based on mutual information rather than Victor–Purpura we used here [0.26 ± 0.000, c.f. Figure 5B vs. 0.29 ± 0.02 in Bettencourt et al. (2007)]. One obvious difference between Random and pattern topologies was the reduced amount of constraint placed on growth patterns. Unlike the patterned topologies, connectivity in the Random networks was *entirely* self determined rather than only partially self determined in patterned networks. Hence, in so-called “random” topologies network structure is based on each neurons own internal properties that at least *in vivo*, have been reported to mediate the formation of structural information (Sporns et al., 2004; Stam and de Bruin, 2004; Achard et al., 2006; van den Heuvel and Pasterkamp, 2008; Massobrio et al., 2015). Known as neural economics, reports from *in vivo* and *ex vivo* assays implicate connectivity may develop to maximize topological efficiency, robustness, modularity, and rich club-like networks composed of network hubs (Bullmore and Sporns, 2012) that may offer advantages in terms of computational performance (Crossley et al., 2013; Senden et al., 2014; Baggio et al., 2015). In fact, recent studies have verified that functional connections of the brain network may be organized in a highly efficient small-world manner *in vivo* (Sporns et al., 2004; Stam and de Bruin, 2004; Achard et al., 2006; van den Heuvel and Pasterkamp, 2008; Vértes et al., 2012) and mirrored among structural information in dissociated neural culture used here (Bettencourt et al., 2007; Downes et al., 2012; Gritsun et al., 2012; Pu et al., 2013; Vincent et al., 2013; de Santos-Sierra et al., 2014; Schroeter et al., 2015).

At higher node degrees, we found an almost universal enhancement in transmission fidelity whether networks were cultured in a Random or a 2D, 4D, or 8D topologies (c.f. Figures 7E,F). Nodes with higher node degrees were also associated with higher clustering coefficients (Figure 8C), and these higher clustering coefficients were in turn, associated with higher estimates of fidelity. This suggests that nodes associated with highly clustered connectivity among its immediate neighbors tend to communicate with higher fidelity than less clustered network structures. However, at the highest clustering coefficients it was the 4D but not 8D network topologies whose transmission fidelity approached that of Random networks (Figures 8D,E, where *C* > 0.4). While higher clustering coefficients (relative to path length) are associated with small-world network topologies, high clustering has also been associated with greater variability in firing dynamics (Litwin-Kumar and Doiron, 2012) and abnormal values associated with neurological disorders such as schizophrenia (Bassett et al., 2008; Liu et al., 2008) and epilepsy (Baccalá et al., 2004; Bernhardt et al., 2011; Varotto et al., 2012). The advantage of 4D over 8D networks in terms of clustering coefficients and its relation to fidelity is also directly opposite to that found for node degree. For node degree (Figures 7E,F) fidelity in 8D networks was now enhanced relative to 4D at both rate and temporal scales among the few nodes with highest node degrees (*k* > 10). It is well known that network’s node degree distribution can have profound effects on network function and efficiency. In a mathematically random network such as those studied by Erdos and Rényi (1960), each connection is present or absent with equal probability. Hence the degree distribution is typically binomial or Poisson for very large graph sizes. In most real-world networks, however, degree distributions are highly skewed, meaning that their distribution appears with a long tail of values that are far above the mean, and denote the presence of a set of highly connected nodes (Hagmann et al., 2008). In our topologies, long-tailed distributions can easily be observed in the 4D, 8D, and Random networks for node degree (Figure 7A), in-degree (Figure 7B), and out-degree (Figure 7C). In fact long-tailed distributions of node degree in networks with power-law degree distributions have been the focus of a great deal of attention in literature and are sometimes referred to as scale-free networks (Barabási and Albert, 1999). Recent years have witnessed a surge in interest in these high degree nodes which are sometimes identified as “hub” neurons due their exceptionally high number of connections and enhanced control over network dynamics in both hippocampal brain slice (Bonifazi et al., 2009; Cossart, 2014) and random topologies we used in this study (Kudoh et al., 2009; Luccioli et al., 2014; Schroeter et al., 2015).

These putative hubs are often identified by their high-node degree, motif participation, betweenness centrality, and local efficiency (Sporns et al., 2007). These neural hubs are thought to play a central role in network communication and information transfer (Sporns et al., 2007; de Reus and van den Heuvel, 2013; van den Heuvel and Sporns, 2013) and may be a part of a more highly interconnected networks (i.e., “rich club”) that have been hypothesized to serve as a network backbone for transmission and integration of information in the brain (van den Heuvel et al., 2012; Towlson et al., 2013; de Reus and van den Heuvel, 2014; Mišić et al., 2014) and reported recently *in vitro* (Yu et al., 2008; Shimono and Beggs, 2014; Timme et al., 2014; Schroeter et al., 2015). These rich club networks may also modulate the dynamical interactions among other lower-degree nodes (Crossley et al., 2013; Senden et al., 2014). The fidelity during communication among nodes was also dependent on the similarity between nodes in terms of node degrees. In other words, degree–degree correlations in which nodes with similar degrees communicated with higher fidelity than those with dissimilar degrees with highest degree nodes communicating at the highest fidelity with other high degree nodes (c.f. Figure 9), at least in the 4D, 8D, and Random topologies.

## Conclusion

In this study, we created four network topologies composed of living cortical neurons that differed in the amount of convergence embedded within the structure of each network. Each network was cultured of a grid of electrodes that permitted high-resolution real-time measurement of neural activity that we used to investigate the relationship between the structure of each network and its functional dynamics. Of topologies we tested, so-called Random networks in which neurons connect based on their own intrinsic properties transmitted information in the form of spike trains with higher fidelity at both rate and temporal coding scales than any other topology we tested. Within the topologies in which we explicitly manipulated structure, the effect of convergence (i.e., node degree) on fidelity was dependent on time-scale with which fidelity was assessed, actual node degree and clustering coefficients, and degree–degree correlations. The effect of node degree depended upon whether that node was highly connected or possessed fewer connections and the degree of the node with which it communicates (i.e., nodes with similar node degrees communicate with higher fidelity across all degree values).

The functional interpretation of the connectome could be a potentially powerful tool toward our understanding of the relationship between structure, function, and brain dynamics in general. While it is possible that structural connectivity may one day enable us to predict function based on structural information alone it is still not clear how those relationships will be established. In this paper, we highlight the role this patterning technology might provide to unravel the complex interaction between network architecture, functional dynamics, and transmission of information.

## Author Contributions

SA, EF, BW, and TD designed the study. SA and EF prepared the cell cultures and collected the data. LP made the molds. SA, TD, and LP analyzed the data. SA, TD, and BW prepared the manuscript. EF, LP, and SL provided critical input for the manuscript. All approve of the final manuscript and accuracy and integrity of the work.

## Conflict of Interest Statement

The authors declare that the research was conducted in the absence of any commercial or financial relationships that could be construed as a potential conflict of interest.
